# Peripheral Reticular Pigmentary Degeneration and Choroidal Vascular Insufficiency, Studied by Ultra Wide-Field Fluorescein Angiography

**DOI:** 10.1371/journal.pone.0170526

**Published:** 2017-01-23

**Authors:** Kunho Bae, Kyuyeon Cho, Se Woong Kang, Sang Jin Kim, Jong Min Kim

**Affiliations:** Department of Ophthalmology, Samsung Medical Center, Sungkyunkwan University School of Medicine, Seoul, Republic of Korea; University of Manchester, UNITED KINGDOM

## Abstract

**Purpose:**

To explore the pathogenesis of peripheral reticular pigmentary degeneration (PRPD) and its clinical significance.

**Methods:**

This cross-sectional, observational study (conducted between January 2010 and May 2015) enrolled 441 eyes of 229 subjects, including 35 eyes with PRPD and 406 eyes without PRPD, which was identified by ultra-wide-field fluorescein angiography (UWFA). The distribution and angiographic circulation time of PRPD were assessed by UWFA. The frequencies of systemic and ophthalmologic comorbidities were compared between groups. Univariate and multivariate generalized estimation equation methods were used to determine the risk factors for PRPD.

**Results:**

The patients with PRPD had a mean age of 75.7 ± 8.5 years (range, 59–93 years), whereas the patients without PRPD had a mean age of 60.1 ± 14.9 years (range, 9–92 years). All eyes with PRPD manifested the lesion in the superior nasal periphery with or without circumferential extension. Among those, only 16 eyes (45.7%) in the PRPD group showed distinctive features in the same location on fundus photographs. There was significant choroidal filling delay in the PRPD group when compared with the control group (1.42±1.22 vs. -0.02±1.05 seconds, *P* < 0.001). Multivariate regression analysis revealed that older age (*P* < 0.001), stroke (*P* = 0.018), ischemic optic neuropathy (*P* < 0.001), and age-related macular degeneration (*P* = 0.022) were significantly associated with PRPD.

**Conclusions:**

UWFA may enhance the diagnostic sensitivity of PRPD. Choroidal vascular insufficiency with compromised systemic circulation in the elderly was related to the manifestation of PRPD. These results help to better understand the pathophysiology of PRPD. Co-existence of systemic and ophthalmic circulatory disorders should be considered in patients with PRPD.

## Introduction

Peripheral reticular pigmentary degeneration (PRPD) is an uncommon, distinct, and clinically significant fundus change, although its nomenclature has changed with increased understanding of the disease [1–5]. PRPD is characterized by a reticular pigmentation that forms a polygonal, netlike arrangement of hyperpigmented lines forming geometric patterns in the peripheral fundus. Although preceding reports noted the major ophthalmologic features of PRPD, understanding of its pathogenesis and clinical importance remains incomplete. This is, in part, because further research on PRPD was limited by available visualization techniques for a considerable period of time.

Since its initial discovery, retinal imaging technology has greatly evolved. Ultra wide-field fluorescein angiography (UWFA) has allowed visualization of many different areas of the retina during angiography and can visualize areas of the peripheral retina that could not previously be photographed [6]. Improved angiographic visualization of the peripheral retina has allowed researchers to learn more about retinal diseases, and, notably, ischemic retinopathy [7,8]. The peripheral location of PRPD, potentially a disease at the chorioretinal juncture, makes UWFA the best modality to visualize these changes.

This study was conducted both to characterize the pathogenesis of PRPD with regard to choroidal perfusion using UWFA, and to illuminate its clinical significance.

## Methods

This was a cross-sectional observational study of PRPD. All investigations adhered to the tenets of the Declaration of Helsinki. This study was approved by the institutional review board of the Samsung Medical Center Patient records were anonymized and de-identified prior to analysis.

One experienced retina specialist (S.W.K.) identified PRPD by extensively reviewing all the UWFA studies of patients who visited the retina clinic of the Samsung Medical Center (Seoul, Korea) between January 2010 and May 2015. All patients with peripheral pigmentary changes demonstrating a coarse, netlike arrangement of pigment lines that formed a polygonal geometric pattern on UWFA were categorized into the PRPD group.

Data of the eyes with these UWFA findings were collected and included in the PRPD group. The patients who underwent UWFA but did not show PRPD findings were assigned to the control group. Subjects in both the PRPD and control groups had under UWFA for the diagnosis of diverse retino-choroidal diseases such as diabetic retinopathy, retinal vascular obstructive diseases, uveitis, and retinal vasculitis. Even subjects in the PRPD group had undergone preferential UWFA examination because of comorbid vitreoretinal diseases, with PRPD as an incidental finding. The patients with bilateral retinal laser scars due to prior pan-retinal photocoagulation or a significant media opacity such as a vitreous hemorrhage or cataract that would preclude acquisition of clear UWFA images were excluded. Thus, the patients who underwent UWFA but did not show PRPD findings were assigned to the control group for comparative analysis.

All patients had undergone ophthalmologic evaluation including anterior segment examination, dilated fundus examination, and UWFA. The presence of age-related macular degeneration (AMD; drusen, geographic atrophy, and neovascular AMD) was assessed by masked grading of color fundus images according to the simplified grading scale of the Age-Related Eye Disease Study (AREDS). Data on demographic review of systemic diseases, ocular comorbidities, sex, age, best corrected visual acuity, refractive error, and blood tests including fasting blood glucose level, post prandial 2-hour blood glucose test, and percentage hemoglobin A1c were collected through electronic records of each patient. Systolic blood pressure and diastolic blood pressure at the time UWFA performed was also collected.

### Ultra-wide field fluorescein angiography

All UWFA studies (Optomap fa plus, Dunfermline, Scotland, UK) were conducted according to a standard protocol after intravenous infusion of 5 ml of 10% sodium fluorescein into one antecubital vein. Images were digitally archived and reviewed using the review software (V2 Vantage, Optos, Dunfermline, UK) allowing full zoom functionality for the review of all images. Images were digitally captured, and subsequently compressed into high-quality Joint Photographic coding Experts Group (JPEG) files.

The quadrant distribution of PRPD was assessed with UWFA images. UWFA images were subdivided into quadrants and hour units to measure the precise extent of lesions.

Arm to choroidal flush time (ACT) and arm to retina time (ART) were assessed in both the PRPD and control groups by a blinded observer. ACT was defined by the interval of time from the completion of injection of fluorescein dye into the antecubital vein to the appearance of the choroidal flush. Early choroidal fluorescence, which appears as a faint, patchy and irregularly scattered distribution of dye throughout the posterior fundus was referred to as the choroidal flush. The ART was measured by noting the initial appearance of fluorescein dye in the central retinal artery. Delayed choroidal filling was defined as the difference between the ACT and ART. Due to the innate properties of the angiography process, ACT and ART were measured in only the eye in which the initial photographs were taken.

### Statistical analysis

Basic characteristics are summarized as the means ± standard deviation for continuous variables and number with percent for categorical variables. To determine risk factors for PRPD, we used univariate and multivariate generalized estimation equation methods that accommodate cluster effects because there are both unilateral and bilateral data. Multivariate analysis was conducted with the variables having a p-value less than 0.05 in the univariate analysis. Statistical analyses were performed by an independent statistician using SAS version 9.4 (SAS Institute, Cary, NC) and R 3.0.3 (Vienna, Austria; http://www.R-project.org). P value less than 0.05 was considered significant.

## Results

A total of 481 eyes of 254 patients were assessed with UWFA. Among the participants, 29 eyes of 16 patients were excluded because of prior pan-retinal photocoagulation, 7 eyes of 5 patients were excluded because of media opacity, and 4 eyes of 4 patients were excluded because of blindness. Thus, 35 eyes of 20 patients (9 males and 11 females) with PRPD and 406 eyes of 209 patients (109 males and 100 females) without PRPD were ultimately included in this study (S1 Data).

**Fig 1:**
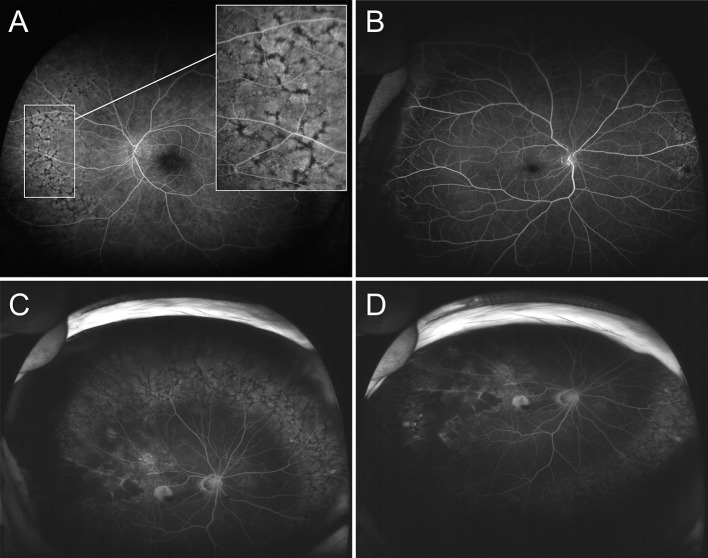
Ultra wide-field angiography (UWFA) of peripheral reticular pigmentary degeneration (PRPD) patients. (Top left) UWFA from the left eye of a patient with underlying diabetes mellitus and hepatocellular cancer. Circumferential pigmentary patches are shown in polygonal, netlike patterns in the nasal peripheral fundus. The contralateral eye demonstrated a symmetric distribution of peripheral pigmentary changes. (Top right) UWFA from the right eye of a patient with an underlying stroke and cerebral aneurysm. There is minimal extension of PRPD to the superior nasal retina. Retinal vein occlusion was identified in the contralateral eye but PRPD was not demonstrated. (Bottom left, right) Another patient with underlying systemic hypertension and retinal vein occlusion. Rather extensive PRPD is found surrounding almost three quadrants of the peripheral retina.

**Fig 2:**
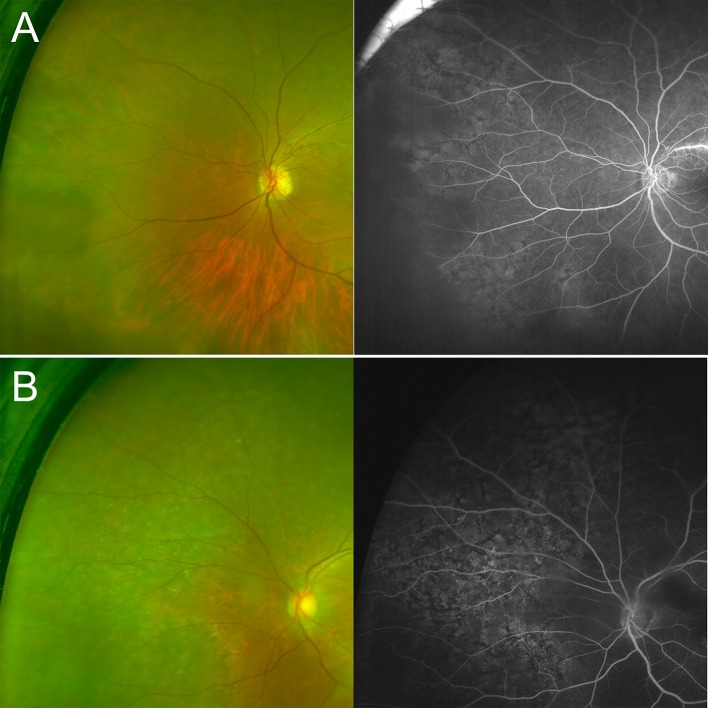
Fundus photography and ultra wide-field angiography of patients with peripheral reticular pigmentary degeneration. (Top column) The peripheral reticular pigmentary change is not obvious in the ultra-wide-field color photograph. (Bottom column) In contrast, the pigmentary change is quite evident in the ultra-wide-field color photograph.

In eyes with PRPD, circumferential reticular pigmentation compartmentalized by linear cracks forming a unique polygonal, netlike pattern was observed (Fig 1). This finding was typically noted near the equatorial fundus. These lesions emerged during the arteriovenous phase of the angiogram, and the pattern was plainly visible with choroidal filling and faded over time. Corresponding funduscopic changes were detected by ultra-wide-field color photography in only 16 eyes (45.7%) among the PRPD patients (Fig 2).

Baseline demographic data of the study population are shown in Table 1. The patients with PRPD had a mean age of 75.7 ± 8.5 years (range, 59–93 years), whereas the patients without PRPD had a mean age of 60.1 ± 14.9 years (range, 9–92 years). And the difference was significant (*P* < 0.001). Laboratory test results including fasting blood glucose level, post prandial 2-hour blood glucose test, percentage hemoglobin A1c, and blood pressure including systolic and diastolic blood pressure was not significantly different between the two groups.

**Table 1:** Baseline characteristics.

Parameters	PRPD group (*n* = 20)	Control group (*n* = 209)	*P*-value
Sex (M / F)	9 / 11	109 / 100	0.542
Age (yrs)	75.7 ± 8.5	60.1 ± 14.9	< 0.001
Glucose, fasting (mg/dl)	126.0 ± 37.8	141.9 ± 45.6	0.138
Postprandial 2-hour blood (mg/dl)	216.5 ± 98.0	205.6 ± 68.9	0.937
HbA1c (%)	7.1 ± 1.4	7.5 ± 1.2	0.216
Systolic blood pressure (mmHg)	130.0 ± 16.1	127.0 ± 18.6	0.525
Diastolic blood pressure (mmHg)	70.1 ± 10.9	71.8 ± 11.3	0.523

PRPD = peripheral reticular pigmentary degeneration; M = male; F = female; yrs = years; HbA1c = percentage hemoglobin A1c.

The list and proportion of ophthalmologic diseases in the PRPD group and control group is shown in Table 2. It included diverse retino-choroidal diseases, for which UWFA was primarily taken. Diabetic retinopathy and retinal vein occlusion patients comprised the majority of the control group.

**Table 2:** Univariate comparison of the frequency of ocular comorbidities.

Parameters	PRPD group (*n* = 35)	Control group(*n* = 406)	*P*-value
BCVA (logMAR)	0.32 ± 0.62	0.23 ± 0.47	0.245
Refractive error, D	0.40 ± 1.28	-0.54 ± 2.12	0.012
Phakia (n = 12/286), D	1.47 ± 0.91	-0.64 ± 2.28	< 0.001
Pseudophakia (n = 23/114), D	0.17 ± 0.74	-0.27 ± 1.61	0.643
Diabetic retinopathy, n (%)	17 (48.6)	188 (46.3)	0.770
Retinal vein occlusion, n (%)	8 (22.9)	66 (16.3)	0.343
Epiretinal membrane, n (%)	9 (25.7)	53 (13.1)	0.045
Uveitis, n (%)	0	18 (4.3)	
Retinal vasculitis, n (%)	0	7 (1.7)	
Capillary hemangioma, n (%)	0	5 (1.2)	
Ocular ischemic syndrome, n (%)	2 (5.7)	3 (0.7)	0.024
Coat’s disease, n (%)	0	3 (0.7)	
Retinal artery occlusion, n (%)	4 (11.4)	2 (0.5)	< 0.001
Retinitis pigmentosa, n (%)	0	2 (0.5)	
Gyrate atrophy, n (%)	0	2 (0.5)	
Ocular albinism, n (%)	0	2 (0.5)	
Hypertensive retinopathy, n (%)	1 (2.9)	1 (0.2)	0.079
Ischemic optic neuropathy, n (%)	3 (8.6)	1 (0.2)	0.002
Degenerative myopia, n (%)	0	1 (0.2)	
Acute retinal necrosis, n (%)	0	1 (0.2)	
AZOOR, n (%)	0	1 (0.2)	
Juvenile retinoschisis, n (%)	0	1 (0.2)	
Choroidal melanoma, n (%)	0	1 (0.2)	
Choroidal metastasis, n (%)	0	1 (0.2)	
Total AMD, n (%)	18 (51.4)	62 (15.3)	< 0.001
Drusen, n (%)	13 (37.1)	45 (11.1)	< 0.001
CGA, n (%)	6 (17.1)	23 (5.7)	0.038
Neovascular AMD, n (%)	0	4 (1.0)	

PRPD = peripheral reticular pigmentary degeneration; BCVA = best corrected visual acuity; AZOOR = acute zonal occult outer retinopathy; AMD = age-related macular degeneration; CGA = geographic atrophy involving the central macula

Duplicate entries were permitted.

The mean logMAR best corrected visual acuity was 0.32 ± 0.62 for the PRPD group, and 0.23 ± 0.47 for the control group (*P* = 0.245). The mean refractive error, expressed as the spherical equivalent, was 1.47 ± 0.91 diopters for the PRPD group and -0.64 ± 2.28 for the control group in phakic eyes (*P* < 0.001), and 0.17 ± 0.74 diopters for the PRPD group and -0.27 ± 1.61 for the control group in pseudophakic eyes (*P* = 0.643).

In terms of univariate analysis for ocular comorbidities, retinal artery occlusion, ischemic optic neuropathy, epiretinal membrane and ocular ischemic syndrome were shown to be significantly associated with PRPD. There was no statistically significant difference between the two groups in terms of the incidence of diabetic retinopathy, retinal vein occlusion, hypertensive retinopathy, uveitis and retinal vasculitis (Table 2). A total of 18 (51.4%) eyes with PRPD had AMD, with either intermediate or large-sized drusen, geographic atrophy involving the central macula, neovascular AMD, or some combination of these. Whereas, 62 (15.3%) eyes showed involvement of AMD in the control group (*P* < 0.001). Drusen (*P* < 0.001) and geographic atrophy involving the central macula (*P* = 0.038) were found to be associated with PRPD (Table 2).

Various cardiovascular and systemic conditions were assessed for an association with PRPD (Table 3). Among them, systemic hypertension was shown to be significantly correlated with PRPD on univariate analysis (*P* < 0.001), whereas diabetes mellitus was not found to have a significant association. Stroke (*P* < 0.001) and carotid artery stenosis (*P* = 0.036) were shown to be significantly associated with PRPD. Otherwise there was no statistically significant difference between the two groups in terms of the incidence of brain aneurysm, cerebral hemorrhage, arrhythmia, myocardial infarction, coronary stenosis, renal disease, and systemic malignancy.

**Table 3:** Univariate comparison of the frequency of systemic comorbidities.

Systemic Disease	PRPD (*n* = 20)	Control (*n* = 209)	*P-*value
Diabetes mellitus, n (%)	10 (50.0)	124 (59.3)	0.421
Hypertension, n (%)	14 (70.0)	78 (37.3)	0.007
Stroke, n (%)	7 (35.0)	6 (2.9)	< 0.001
Carotid artery stenosis, n (%)	4 (20.0)	14 (6.7)	0.036
Cerebral hemorrhage, n (%)	0	4 (1.9)	0.926
Brain aneurysm, n (%)	1 (5.0)	3 (1.4)	0.257
Arrhythmia, n (%)	0	5 (2.4)	0.977
Myocardial infarction, n (%)	0	2 (1.0)	0.692
Coronary artery stenosis, n (%)	0	13 (6.2)	0.515
Chronic renal disease, n (%)	2 (10.0)	18 (8.6)	0.778
Systemic malignancy, n (%)	4 (20.0)	20 (9.6)	0.130

PRPD = peripheral reticular pigmentary degeneration.

Duplicate entries were permitted.

In multivariate logistic regression analysis, age (*P* = 0.024), stroke (*P* = 0.018), refractive error (*P* = 0.043), ischemic optic neuropathy (*P* < 0.001), and AMD (*P* = 0.022) were the significant factors that correlated with PRPD (Table 4). Retinal artery occlusion and ocular ischemic syndrome showed marginal significance. Additional age-matched analysis was performed since age is potentially such a strong confounder for most of the proposed factors (S1 Table). In consequence, stroke, drusen, and age-related macular degeneration were again the significant factors that correlated with PRPD.

**Table 4:** Multivariate logistic regression analysis to identify factors correlated with peripheral reticular pigmentary degeneration.

Variables	P-value	OR (95% CI)
Age	<0.001	1.120 (1.015–1.235)
Systemic hypertension	0.584	0.700 (0.195–2.512)
Stroke	0.018	7.529 (1.424–39.801)
Carotid artery stenosis	0.895	1.124 (0.198–6.381)
Refractive error	0.043	1.300 (1.009–1.676)
Lens status	0.147	2.522 (0.722–8.812)
Retinal Artery Occlusion	0.080	43.471 (0.638–2960.460)
Epiretinal membrane	0.728	1.261 (0.341–4.663)
Ischemic optic neuropathy	<0.001	82.641 (6.482–1053.739)
Ocular ischemic syndrome	0.051	5.430 (0.995–29.645)
AMD	0.022	3.999 (1.221–13.099)

AMD = age-related macular degeneration.

Among 20 patients diagnosed with PRPD, 15 of the 17 patients (88.2%) with available images showed bilateral involvement of PRPD. Only two subjects showed no contralateral eye involvement of PRPD despite the acquisition of clear UWFA images. In these two patients, only a minimal extent of PRPD was observed in the affected eyes.

**Fig 3:**
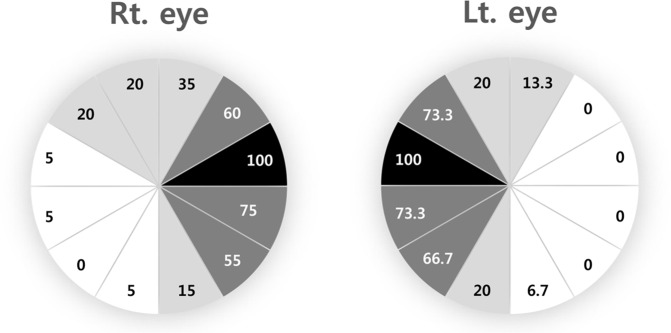
Clockwise frequencies (%) of peripheral reticular pigmentary degeneration (PRPD) lesions among the eyes of the PRPD group are illustrated. The superior nasal quadrant of the fundus was mostly involved in both eyes. Temporal involvement is rare.

There was symmetry in the distribution of PRPD lesions between the eyes of each patient. All of the eyes with PRPD manifested the lesion at least in the superior nasal peripheral fundus, particularly between the 2–3 o’clock area in the right eye and the 9–10 o’clock area in the left eye. There was no case of PRPD exclusively confined to the temporal quadrant. The pigmentary patch was continuous and distributed circumferentially from the nasal aspect of the peripheral retina. The distribution frequency of PRPD is illustrated in Fig 3. The extent of PRPD varied from minimal, localized only to the nasal quadrant, to extensive with almost 270 degrees of involvement of the peripheral retina (Fig 1). All of the lesions were discovered in the peripheral retina, and posterior pole involvement was not noted.

The circulation time measured with UWFA is shown in Table 5. In the control group, 8 patients with insufficient initial photographs to determine ART and ACT were excluded. The mean ACT was 11.85 ± 5.25 seconds for patients with PRPD and 10.14 ± 4.12 seconds for the control group (*P* = 0.113), whereas the mean ART was 10.47 ± 5.52 seconds for patients with PRPD and 10.17 ± 4.14 seconds for the control group (*P* = 0.958). Delayed choroidal filling time, that is, the difference between the ACT and ART, was 1.42 ± 1.22 seconds for the patients with PRPD and -0.02 ± 1.05 seconds for the control group (*P* < 0.001). Consequently, the regular sequence of filling of the retinal arteries following the choroidal flush tended to be reversed in PRPD patients.

**Table 5:** Angiographic circulation time in the peripheral reticular pigmentary degeneration (PRPD) and control group.

Parameters	PRPD (*n* = 20)	Control (*n* = 209)	*P*-value
Arm to choroidal time, seconds	11.85 ± 5.25	10.14 ± 4.12	0.113
Arm to retina time, seconds	10.47 ± 5.52	10.17 ± 4.14	0.958
Delayed choroidal filling, seconds	1.42 ± 1.22	-0.02 ± 1.05	< 0.001

## Discussion

In this study, bilateral continuous circumferential pigmentary changes in the peripheral retina were identified by UWFA and denominated as PRPD. PRPD has been described under several appellations such as reticular pigmentary degeneration, senile reticular pigmentary degeneration, reticular degeneration of the pigment epithelium, senile peripheral pigmentary degeneration or peripheral reticular pigmentary change [1–5]. Although postmortem studies have reported a considerable incidence of PRPD in Caucasians [9], yet there has been no report of PRPD in an Asian population. There could be interracial differences in its prevalence. However, the dissimilarity of detection sensitivity related to the fundus pigmentation could also be another explanation. About half of the cases of PRPD diagnosed by UWFA showed no clearly distinguishable reticular pigmentary changes on ultra-wide-field color photography. The result implies that UWFA is a sensitive measure to identify PRPD, at least, in pigmented people.

A prospective controlled study would be impractical, since this entity was discovered rather infrequently. Instead, a consecutive series of patients who underwent UWFA for various retino-choroidal diseases were set as a control group and used for comparative analysis.

Of note, multivariate regression analysis revealed that stroke was found to be significantly correlated with the incidence of PRPD. This may imply the role of systemic circulatory disorders or vascular insufficiency in the pathogenesis of PRPD. In addition, ischemic optic neuropathy was also found significantly more often in PRPD patients, and ophthalmic diseases relevant to impaired ocular circulation such as retina arterial occlusion, ocular ischemic syndrome were marginally correlated. These results suggest the relationship between PRPD and systemic, ophthalmic circulatory disorders. That is, PRPD is a novel peripheral pigmentary change for which the possibility of specific systemic or ocular comorbidity might be considered.

The current study also shows that angiographic circulation time tends to be prolonged in PRPD patients. Choroidal circulation was more delayed than retinal circulation. Because the choroid usually begins to fluoresce 1 or 2 seconds before the initial filling of the central retinal artery [10], the normal sequence of retinal arteriole filling following the choroidal flush was reversed in subjects with PRPD. This suggests that the association of PRPD and derangement in choroidal circulation should be taken into account.

Demographic characteristics of patients were analyzed to identify common characteristics and to understand the nature and developmental mechanisms of PRPD. Although the extent of involvement was variable, all of the lesions were limited to the peripheral retina and most showed bilateral involvement. The choroid is supplied by two arterial systems; the posterior choroid by the short posterior ciliary arteries, and the anterior choroid by the peripheral retrograde choroidal arteries, which arise from the major arterial circle of the iris, the long posterior ciliary arteries, or the anterior ciliary arteries [11,12]. The presence of a watershed zone has been reported between the anterior and posterior choroidal arteries, which lies in the equatorial region circumferentially [12,13]. Thus, the peripheral choroid near the watershed zone may be susceptible to impaired perfusion [14]. While reporting the entity of ‘equatorial reticular pigmentary degeneration,’ Hayreh proposed the possible pathogenic mechanism of the entity to be chronic choroidal ischemia in the equatorial watershed zone [13,15]. In addition, a polygonal, netlike pattern of the PRPD could be related to the lobular structure of choriocapillaris in the equatorial watershed zone. Multivariate analysis also revealed that aging was an independent risk factor for PRPD. Ito and associates reported that choroidal arterioles in subjects of advanced age filled more slowly and became more sparse with less branching than younger subjects [16]. This could be a possible explanation for the preponderance of PRPD in elderly patients. Besides, refractive error was found to be correlated with the incidence of PRPD. Significant hyperopia was one of the interesting features of PRPD patients, raising doubts about the anatomical predisposition of PRPD in short axial length. There is a possibility that the incidence of choroidal insufficiency varies according to the axial length as the choroidal thickness varies depending on the axial length. However, the majority of patients had not undergone axial length examination, and this is one of the limitations of our study. Further study on this issue would be meaningful.

It is worth noting that all of the eyes with PRPD manifested the lesion in the superior nasal peripheral fundus, with occasional extension of the lesion circumferentially to the temporal retina. There were also no cases of PRPD presenting exclusively in the temporal periphery. These results are corroborated by previous reports showing a predilection for the nasal and superior quadrant of the fundus [2,9]. Feke and associates reported that blood flow to the temporal side of the retina was approximately three times larger than to the nasal side [17]. The blood flow to the inferior retina was 6% greater than the flow to the superior retina, although this was not statistically significant. Choroidal circulation is easily affected by the amount of blood flow since it is an unusually reactive, high flow system and supplies the outer retina by long-range diffusion [18]. This suggests the vulnerability of the superior nasal choroidal circulation and may provide a basis for the prevailing involvement of PRPD in the nasal and superior periphery. Thus, from a clinical perspective, the superior nasal peripheral section of the retina should be scrutinized with attention when reviewing fluorescein angiographic images.

Based on observations in the current study, we speculate that PRPD may occur due to ischemic choriocapillaris secondary to choroidal vascular insufficiency in conjunction with compromised systemic circulation. The degenerative change may start in the superior nasal retina, which is mostly vulnerable to choroidal vascular insufficiency.

Peripheral lesions such as lattice degeneration, paving stone degeneration and crystalline retinopathy should be included in the differential diagnosis of PRPD [19]. There have been anecdotal reports of peripheral pigmentary fundus changes characterized by extensive cobblestone degeneration extending into the mid-periphery, or wedge-shaped peripheral degeneration in carotid occlusive disease and ocular ischemic syndrome [20]. In addition, pattern dystrophy and Sjögren reticular dystrophy could also be included in the differential diagnosis, but there is a difference in the mean age of the patients with these diagnoses, and the characteristic macular pigmentary changes are usually absent [21,22]. The differential diagnosis may also include congenital grouped albinotic retinal pigment epithelial spots, retinitis pigmentosa, and pigmentary changes secondary to trauma or inflammation [1,9,23–26].

Previous reports indicated that about a third of patients with age-related macular degeneration manifested PRPD [27], and two thirds of patients with PRPD have accompanying age-related macular degeneration [2]. The current study also confirmed a significant association between AMD and PRPD. This result indicates that PRPD is related to AMD and may suggest that AMD changes are not limited to the macula. Moreover, a shared genetic risk between PRPD and AMD was noted, since a recent study has identified correlations between PRPD and a complement factor H variant [5]. Choroidal ischemia had also been frequently noted to be related to the development of AMD [28,29]. PRPD, which shares genetic risk with AMD, is thought to promote manifestations of AMD on account of its relation to choroidal insufficiency. In turn, the present study indirectly supports the previous hypothesis that AMD is triggered by choroidal ischemia in patients with susceptible genes [30].

Distinct angiographic findings of PRPD are interesting to note; however, more importantly, its systemic and ocular associations should be taken into account. When these features are identified, physicians should consider the possibility of systemic comorbidities such as stroke and ophthalmologic comorbidities such as ocular ischemic syndrome, ischemic optic neuropathy, and AMD. Thus, the identification of PRPD may necessitate surveillance and patient education in this regard.

There were several limitations to the present study. First, the retrospective nature and the relatively small number of participants might have limited the statistical power to support our statement. In addition, due to the rarity of the disease entity, the duration of this cross-sectional study was inevitably prolonged. Second, there may be statistical bias due to the manner in which the control group was created. A heterogeneous control group may have masked some factors affecting the development of PRPD. Third, ultra-wide-field color photographs produced using the false color image technique were used to compare the corresponding angiographic findings. Fourth, results of histologic investigation and supplemental ophthalmologic tests other than UWFA were not available in the majority of eyes. For example, optical coherence tomography and electroretinograms of the lesions could have offered more information about PRPD. In spite of these, the present study addresses the relationship between PRPD and choroidal insufficiency by measuring actual ocular circulation time and investigating systemic and ocular comorbidities.

In conclusion, the diagnostic sensitivity of PRPD can be improved by UWFA imaging. Compromised systemic circulation and choroidal vascular insufficiency in the elderly may play a pathogenic role in the development of PRPD.

## Supporting Information

S1 Data Dataset of 229 patients who underwent ultra-wide-field fluorescein angiography for the diagnosis of diverse retino-choroidal diseases. (XLSX)

S1 Table Age-matched analysis of systemic and ocular comorbidities in peripheral reticular pigmentary degeneration. (DOCX)
