# Supplementary material for: Peripheral Reticular Pigmentary Degeneration and Choroidal Vascular Insufficiency, Studied by Ultra Wide-Field Fluorescein Angiography
Source: PLoS One. 2017 Jan 23;12(1):e0170526. doi: 10.1371/journal.pone.0170526 (PMC5256899; doi:10.1371/journal.pone.0170526)
Supplement: S1 Table — (DOCX) [file pone.0170526.s002.docx]

**S1 Table.** Age-matched analysis of systemic and ocular comorbidities in peripheral reticular pigmentary degeneration.

| **Variables** | **Univariate *P-*value** | **Multivariate**  ***P-*value** | **OR (95% CI)** |
| --- | --- | --- | --- |
| Systemic comorbidities |  |  |  |
| Sex | 0.8113 |  |  |
| Diabetes Mellitus | 0.1524 |  |  |
| Systemic hypertension | 0.1568 |  |  |
| Stroke | 0.0026 | 0.0126 | 26.677(2.022-352.024) |
| Carotid artery stenosis | 0.1032 |  |  |
| Chronic renal disease | 0.2127 |  |  |
| Systemic malignancy | 0.7075 |  |  |
| Ocular comorbidities |  |  |  |
| Spherical equivalent | 0.9668 |  |  |
| Lens status | 0.1954 |  |  |
| Diabetic retinopathy | 0.7249 |  |  |
| Retinal vein occlusion | 0.2618 |  |  |
| Epiretinal membrane | 0.5853 |  |  |
| Hypertensive retinopathy | 0.9237 |  |  |
| Glaucoma | 0.8859 |  |  |
| Ocular ischemic syndrome | 0.1199 |  |  |
| AMD | 0.0160 | 0.0820 | 7.244(0.778-67.491) |
| Drusen | 0.0347 | 0.6334 | 0.605(0.077-4.775) |
| CGA | 0.3934 |  |  |
| Angiographic circulation time |  |  |  |
| ART | 0.6458 |  |  |
| ACT | 0.5474 |  |  |
| DCF | 0.0001 |  |  |

PRPD=peripheral reticular pigmentary degeneration; AMD=age-related macular degeneration; CGA=geographic atrophy involving the central macula; ART= arm to retina time; ACT= arm to choroid time; DCF= delayed choroidal filling

Duplicate entries were permitted.

Multivariate analysis was conducted with the variables having a p-value less than 0.05 in the univariate analysis.
